# Microwave-Assisted Hydrothermal Treatment of Multifunctional Substituted Hydroxyapatite with Prospective Applications in Bone Regeneration

**DOI:** 10.3390/jfb14070378

**Published:** 2023-07-19

**Authors:** Alexandra-Cristina Burdusel, Ionela Andreea Neacsu, Alexandra Catalina Birca, Cristina Chircov, Alexandru-Mihai Grumezescu, Alina Maria Holban, Carmen Curutiu, Lia Mara Ditu, Miruna Stan, Ecaterina Andronescu

**Affiliations:** 1 Department of Science and Engineering of Oxide Materials and Nanomaterials, Faculty of Chemical Engineering and Biotechnologies, University Politehnica of Bucharest, 1–7 Gheorghe Polizu Street, 011061 Bucharest, Romania; 2 Academy of Romanian Scientists, Splaiul Independentei 54, 050044 Bucharest, Romania; 3 Research Institute of the University of Bucharest—ICUB, University of Bucharest, 050657 Bucharest, Romania; 4 Department of Microbiology and Immunology, Faculty of Biology, University of Bucharest, 077206 Bucharest, Romania; 5 Department of Biochemistry and Molecular Biology, Faculty of Biology, University of Bucharest, 050095 Bucharest, Romania

**Keywords:** hydroxyapatite, magnesium, cerium, bone regeneration

## Abstract

Orthopedic bone graft infections are major complications in today’s medicine, and the demand for antibacterial treatments is expanding because of the spread of antibiotic resistance. Various compositions of hydroxyapatite (HAp) in which Calcium (Ca^2+^) ions are substituted with Cerium (Ce^3+^) and Magnesium (Mg^2+^) are herein proposed as biomaterials for hard tissue implants. This approach gained popularity in recent years and, in the pursuit of mimicking the natural bone mineral’s composition, over 70 elements of the Periodic Table were already reported as substituents into HAp structure. The current study aimed to create materials based on HAp, Hap-Ce, and Hap-Mg using hydrothermal maturation in the microwave field. This route has been considered a novel, promising, and effective way to obtain monodisperse, fine nanoparticles while easily controlling the synthesis parameters. The synthesized HAp powders were characterized morphologically and structurally by XRD diffraction, Dynamic light scattering, zeta potential, FTIR spectrometry, and SEM analysis. Proliferation and morphological analysis on osteoblast cell cultures were used to demonstrate the cytocompatibility of the produced biomaterials. The antimicrobial effect was highlighted in the synthesized samples, especially for hydroxyapatite substituted with cerium. Therefore, the samples of HAp substituted with cerium or magnesium are proposed as biomaterials with enhanced osseointegration, also having the capacity to reduce device-associated infections.

## 1. Introduction

The main components of a mineralized bone matrix include a calcium phosphate crystal, hydroxyapatite, and various organic materials [1]. Hydroxyapatite (HAp, Ca_10_(PO_4_)_6_(OH)_2_) is among the most promising crystalline calcium phosphate considered to be biomaterial due to its chemical resemblance to the inorganic part of human hard tissues such as bone and teeth [2,3,4]. Stoichiometric and pure HAp crystallizes in the monoclinic system, and it most often crystallizes in the hexagonal system at over 250 °C and thermally decomposes at temperatures between 800 and 1200 °C [5,6]. Bone hydroxyapatite has a small dimensional structure, about 25 nm wide, 50 nm long, and 10 nm high [7]. Due to their quicker growth and development, small microcrystals are favorable for bone regeneration and repair when rapid mineralization is needed, as well as for postnatal and embryonic bone development. In addition, these tiny nanoscale crystals have a high surface area-to-volume ratio and are quickly dispersed by the fluid around them in response to particular circumstances. In the course of homeostatic bone remodeling, the particular crystalline shape of bone hydroxyapatite may promote effective bone demineralization and resorption by osteoclasts [8,9,10].

Hap and other calcium phosphates have long been investigated as biological agents for the treatment and diagnosis of bone diseases due to their qualities such as biocompatibility, osteoconductivity, similarity to the bone mineral phase, and osteogenic activity that ensure a high affinity of these materials for bone tissues [11,12,13]. This affinity is being studied intensively to develop specific delivery systems of biologically active molecules for bone metastases, osteosarcoma, and other bone diseases [14], enabling theranostic applications. In addition, by functionalizing the hydroxyapatite surface with some specific molecules, more accurate delivery to bone tissues is possible [15,16,17,18].

The use of metal ions can be an appropriate alternative for improving HAp because they have high stability and broad antibacterial spectra [19,20]. Magnesium represents a very innovative option for the development of antibacterial bone grafts, as it shows powerful antibacterial activity in certain doses and is highly biocompatible [21,22,23]. The introduction of magnesium into the synthesis reduced bacterial development and biofilm formation in hydroxyapatite-based bone substitutes [13,14]. Moreover, the materials in their composition, magnesium ions, are beneficial for bone regeneration mechanisms, as this compound hastens the healing of bone defects by improving the differentiation and proliferation of osteoblasts [24]. Due to its mechanical and osteopromoting qualities, magnesium (Mg)-based biomaterials have been employed as orthopedic implants for a very long time [25,26,27]. Additionally, their benefits over comparable standard products have been thoroughly studied, with Mg-based alloys, bioceramics [28], bioglass [29], and polymer composites [30], all demonstrating unique superiority in hastening bone formation and fracture healing [31]. Mg-based bioceramics may provide various advantages as biodegradable bone graft alternatives since they can progressively break down and be replaced by fresh bone [32]. Magnesium incorporation for Mg-based polymeric materials not only overcomes acid degradation products but also enhances osteopromoting activity [33]. According to earlier research, magnesium ions (Mg^2+^) have an impact on the pace at which calcium phosphate crystallizes off the surface of bones and the subsequent development of hydroxyapatite [34,35,36]. Additionally, the usage of degradable magnesium metals and alloys produces osteogenic differentiation and osteoblast development, which is induced by Mg^2+^ and promotes bone regeneration [37]. On the other hand, Mg^2+^ shortage (about 0.04–10%) inhibits bone formation by reducing osteoblasts and bone volume and increasing osteoclastic bone resorption as a result of increased proinflammatory cytokine release [38,39].

Cerium, a different metal ion with antibacterial properties, can reduce cytokine levels, decrease inflammation, and give cellular protection in vitro and in vivo, which implies that it may have anti-inflammatory effects on designed tissues. Among the various calcium cationic substitution ions in the hydroxyapatite structure, the Ce ion substitution has been actively investigated [40,41], with HAp-Ce(III) being reported to exhibit silver-like bacteriostatic properties [42,43,44]. Recently, cerium has also been found to have multi-enzymatic properties that make it attractive for biological applications. The biological effects of cerium oxide have been studied, and it has been demonstrated that by directly altering the levels of oxygen in intracellular settings, it is capable of inducing angiogenesis. But it can induce local inflammatory reactions when using a high concentration (over 3.5% CeO_2_) [45,46]. Since the electronegativity and radius of Ce^3+^ are 1.06 and 0.107 nm, respectively, close to those of Ca^2+^ ion (1.01 and 0.100 nm), it can displace Ca^2+^ and penetrate the HAp network to form Ce^3+^ substituted HAp (Ce-HAp), that may result in increased solubility, which may, enhance the antibacterial effect and biodegradability [47]. Compared to other metal ions, Ce^3+^ ions have shown great results as antibacterial agents used in medicine for a long time due to their wide range of antibacterial activity (*Escherichia coli*, *Salmonella typhimurium*, *Bacillus subtilis*, and *Enterococcus faecalis*) [48]. Cerium-based biomaterials represent a new element in research due to the low level of Ce^3+^ ions that do not harm human cells, as well as due to a long-lasting biocide and excellent thermal stability [49].

To increase and improve the range of possible uses for functional nanomaterials, it is crucial to create quick, easy, and environmentally friendly synthetic methodologies [50]. In the present work, hydroxyapatite substituted with cerium and magnesium was obtained through an unconventional, microwave-assisted hydrothermal method. This route has been recently considered a novel, promising, and effective way to obtain monodisperse, fine nanoparticles while easily controlling the synthesis parameters (e.g., initial pressure in the autoclave, the maximum temperature, and heating rate, time). In comparison with the traditional hydrothermal method, the microwave-assisted improved version has several advantages, with great impact on the final material’s properties: (i) homogenous heating in the entire volume of the reaction mixture, without temperature gradients leading to non-homogeneity in particle size and morphology; (ii) rapid heating, due to the microwave irradiation of the polar molecules normally used in the chemical reactions (water, alcohol); (iii) reduced reaction time and energy consumption. As a result, the obtained HA powders have reduced particle size, increased purity, and a narrower size distribution compared with other methods [51,52].

## 2. Materials and Methods

### 2.1. Reagents and Chemicals

The chemicals used to accomplish the experiments are as follows: (NH_4_)_2_HPO_4_ (98%) from Sigma–Aldrich, Ca(NO_3_)_2_*4H_2_O (99%), NH₄OH (25% solution), Ce(NO_3_)_3_*6H_2_O (99%), Mg(NO_3_)_2_*6H_2_O (99%) from Fluka—Honeywell Research Chemicals, and ultrapure water.

### 2.2. Pristine Hydroxyapatite Synthesis

The synthesis of hydroxyapatite was carried out by co-precipitation followed by hydrothermal maturation in a microwave field using the synthWAVE equipment (Milestone Srl, Sorisole, Bergamo, Italy). The operating principle of the equipment is as follows: the reagents are poured into the vials on a rotating support, which will be closed tightly using Teflon caps. The second step is the automatic lowering of the mechanical stirrer. The vials are positioned in a liquid capable of absorbing microwaves (usually water), thus allowing the transfer of energy and heat to the samples. The chamber containing the samples is automatically clamped and pressurized using nitrogen to prevent the boiling of the solvents. At the end of the reaction, an integrated cooling device rapidly lowers the temperature in the chamber where the vials are located. The benefits of the method are the ease of the method and the short synthesis time, the reproduction of the conditions developed in the small-scale reactions, and the modification of the parameters easily.

Calcium nitrate tetrahydrate and diammonium phosphate acid powders, corresponding to a Ca/P ratio of 1.67 (specific to HAp), were solubilized in distilled water under magnetic stirring. The phosphate precursor was added dropwise over the Ca(NO_3_)_2_*4H_2_O, maintaining magnetic stirring. Since the conditions for obtaining hydroxyapatite involve an alkaline medium, the pH of the reaction mixture was monitored and adapted to the range of 10–10.5 by dropping ammonium hydroxide solution 25 wt.%. After reaching this value, the mixture was poured into the containers of microwave-assisted equipment and subjected to hydrothermal maturation. To study the effect of this treatment on the obtained materials, the temperature was varied as a reaction parameter. Thus, a previously obtained mixture was subjected to treatment at 150 ℃, respectively another at 250 ℃, at an initial pressure of 2 bars for 30 min in both cases. 

After this step, the hydroxyapatite was separated by filtration and washed with distilled water until a neutral pH was fulfilled. Finally, the resulting precipitates were dried in an oven at 60 ℃ for 24 h, and the resulting materials were further referenced according to the varied synthesis parameter (temperature), as follows: HAp_150, respectively HAp_250.

### 2.3. Substituted Hydroxyapatite Synthesis

In the case of the synthesis of hydroxyapatite substituted with cerium (Ce^3+^) or magnesium (Mg^2+^) ions, the process is similar to the one mentioned above for obtaining HAp. The new factor involved in this process is represented by the addition of the substituents precursors—Ce(NO_3_)_3_*6H_2_O or Mg(NO_3_)_2_*6H_2_O, corresponding to different Ca^2+^ substitution molar ratio (1, 3, and 5%) in the initial mixture of reaction, along with Ca(NO_3_)_2_*4H_2_O.

Each time, two solutions were prepared, the first resulting from the mixture of Ca(NO_3_)_2_*4H_2_O with 200 mL of distilled water and the stoichiometric amount of Ce/Mg precursor, and the second solution from the mixture of diammonium acid phosphate (NH_4_)_2_HPO_4_ with 200 mL of distilled water. The next step consisted in dropping the second solution over the first solution under magnetic stirring, and the mixture thus obtained was subjected to hydrothermal maturation in the microwave field. In this case, the temperature of 150 °C was chosen based on the characterization results of pristine HAp (although HAp_150 presented a considerably reduced crystallinity, the average crystallite size proved to be lower, an advantage in the case of bone regeneration materials).

The obtained precipitate was filtered, washed with distilled water to reduce the pH of 10 to a physiological one of 7–7.5, and then was left to dry in an oven at 60 ℃ for 24 h. The resulting materials are further referenced according to the varied synthesis parameters (substituent and concentration), as follows: HAp_Ce_1%, HAp_Ce_3%, HAp_Ce_5%, HAp_Mg_1%, HAp_Mg_3%, HAp_Mg_5%.

### 2.4. Morphological and Structural Characterization

#### 2.4.1. X-ray Diffraction

In order to determine the individual crystalline phases that made up the produced powders, the X-ray diffraction technique was used for analysis. In order to complete this, a PANalytical Empyrean diffractometer from Malvern PANalytical in Bruno, The Netherlands, was employed. It was outfitted with a hybrid monochromator (2xGe 220) on the incident side and a parallel plate collimator set on a PIXcel 3D detector on the diffracted side. At room temperature, with an incidence angle of 0.5° for Bragg angle values of 2 between 10° and 80°, an acquisition period of 255 s, a step of 0.01414°, and Cu K radiation with an angle of incidence of 1.5406° (40 mA and 45 kV), X-ray diffraction measurements analysis were accomplished. In order to ascertain the average crystallite size, unit cell parameters, and crystallinity of the examined powders, the diffractograms were further submitted to Rietveld refinement using the HighScore Plus program (version 3.0e, PANalytical B.V. Almelo, The Netherlands). The diffractogram fits were deemed acceptable if the goodness of fit was lower than 4.

#### 2.4.2. Dynamic Light Scattering (DLS) and Zeta Potential

Each series of hydroxyapatite nanoparticles synthesized using different parameters of microwave-assisted hydrothermal treatment and/or substituted with cerium and magnesium ions at different concentrations was characterized by dynamic light scattering (DLS) technique and zeta potential in order to establish the hydrodynamic diameter and surface charges. The measurements were made using the DelsaMax Pro light scattering analyzer (Backman Coulter, Brea, CA, USA), having as a component a laser with a wavelength of 532 nm. The powders were distributed in ultrapure water at a concentration of 0.33 mg/mL at room temperature. To achieve a homogeneous dispersion, all samples were subjected to ultrasound for 10 min using an ultrasound bath.

#### 2.4.3. Scanning Electron Microscopy (SEM)

The QUANTA INSPECT F50 scanning electron microscope (Thermo Fisher, Eindhoven, The Netherlands) was used for the scanning electron microscopy (SEM). It is equipped with an energy-dispersive X-ray spectrometer (EDS) with a resolution at MnK of 133 eV and a field emission electron gun (FEG) with a resolution of 1.2 nm. The powdery samples were set on slide supports with the use of carbon tape in order to analyze the shape and size; they were then placed into the microscope analysis chamber, where the images were captured by recording the ensuing secondary electrons.

#### 2.4.4. Fourier-Transform Infrared Spectroscopy (FT-IR)

Thermo Nicolet’s Nicolet 6700 (Thermo Fisher Scientific, Waltham, MA, USA) FT-IR spectrometer’s ZnSe crystal was used to evaluate a small sample of powder in order to look into the functional groups that are typical of the produced nanoparticles. The analysis was completed at room temperature using 32 scans of the material at a resolution of 4 cm^−1^ between 4000 and 1000 cm^−1^. It was able to record the data obtained by connecting the spectrometer to a data collection and processing device using the Omnic work program. (Thermo Nicolet, Version 8.2).

### 2.5. In Vitro Interactions with Osteoblast Cells

In full Dulbecco’s Modified Eagle’s Medium supplemented with 10% fetal bovine serum, mouse osteoblasts MC3T3-E1 were cultured at 37 °C in a humidified environment with 5% CO_2_. At a cell density of 5 × 104 cells per cm^2^, the cells were planted in 96-well plates and allowed to adhere for the night. These were then sterilized under UV irradiation and incubated with 25, 100, and 250 g/mL HAap samples over the following 24 or 72 h. All in vitro tests were conducted using untreated cells as a control.

#### 2.5.1. Cell Viability Assay

The cellular proliferation was measured using the 3-(4,5-dimethylthiazol-2-yl)-2,5-diphenyltetrazolium bromide (MTT; Sigma-Aldrich, Saint Louis, MO, USA) assay based on the succinate dehydrogenase mitochondrial activity in the viable cells. After 24 and 72 h of incubation, the culture medium was removed, and the cells were incubated with 1 mg/mL MTT for 2 h in the incubator at 37 °C. The purple formazan crystals formed in the viable cells were dissolved with 2-propanol (Sigma-Aldrich, Saint Louis, MO, USA), and the absorbance was measured at 595 nm using FlexStation 3 multi-mode microplate reader from Molecular Devices (San Jose, CA, USA).

#### 2.5.2. Griess Assay

Nitric oxide (NO) content in the culture medium that had been previously collected after 24 and 72 h of incubation was measured using the Griess reagent, a stoichiometric solution (*v/v*) of 0.1% naphthyl ethylenediamine dihydrochloride and 1% sulphanilamide in 5% H_3_PO_4_. Enhanced NO levels are associated with cytotoxic effects because they are directly linked to inflammatory and apoptotic processes. The FlexStation 3 multi-mode microplate reader was used to quantify the absorbance of the solutions as they were acquired at 550 nm. The NaNO_2_ standard curve was used to compute the NO concentration.

#### 2.5.3. Fluorescence Microscopy

Following each incubation period, the osteoblasts were permeabilized for an hour using 0.1% Triton X-100/2% bovine serum albumin (BSA) and fixed with 4% paraformaldehyde for 20 min. The nuclei were counterstained with 2 g/mL DAPI (4′,6-diamino-2-phenylindole) after the actin filaments had been stained with 10 g/mL phalloidin-FITC (fluorescein isothiocyanate). The Olympus IX71 inverted fluorescent microscope (Olympus, Tokyo, Japan) was used to study the cells.

### 2.6. Microbiological Evaluation

The ATCC strains used for this study are *Staphylococcus aureus*, *Enterococcus faecalis* (Gram-positive), *Escherichia coli*, *and Pseudomonas aeruginosa*) (Gram-negative), and yeast (*Candida albicans*) were obtained from the strain collection of the Microbiology laboratory, Faculty of Biology, University of Bucharest.

#### 2.6.1. MIC (Minimum Inhibitory Concentration) Method

A quantitative approach based on serial binary microdilutions in a liquid medium (simple broth), uniformly distributed in 96-well plates, was utilized to calculate the MIC. An amount of the bioactive chemical or nanosystem equal to a concentration of 5 mg/mL was applied to the first well of each row. A micropipette was then used to make 10 binary dilutions, starting with well 1 (concentration of 5 mg/mL) and ending with well 10 (where the ultimate concentration will be 0.009765625 mg/L). Wells 11 and 12 were then used as positive control (medium containing bacteria) and negative control (medium devoid of bacteria), respectively. A total of 15 L of 0.5 McFarland density microbial suspension were then added to each well following the microdilutions. The seeded plates were incubated for 24 h at 37° Celsius, and following incubation, the MIC value for each compound or nanosystem was determined macroscopically as the point at which microbial growth or the emergence of turbidity in the environment was no longer visible [53].

#### 2.6.2. Development of Monospecific Biofilms

A quantitative approach based on the execution of binary serial microdilutions in a liquid medium (simple broth) dispersed sterile in 96-well plates was used to determine the impact of the acquired biomaterials on the development of biofilms. An amount of the bioactive chemical or nanosystem equal to a concentration of 5 mg/mL was applied to the first well of each row. Later, 12 binary dilutions were performed using a micropipette, commencing with well 1 (concentration 5 mg/mL) and ending with well 10 (final concentration 0.009765625 mg/mL). A total of 15 L of 0.5 McFarland density microbial suspension were then added to each well following the microdilutions. The seeded plates were incubated for 24 h at 37 degrees Celsius. Following incubation, the biofilms were thoroughly washed three times with sterile physiological water (AFS) and fixed for 5 min with cold methanol.

The dry plates were dyed with 1% crystal violet solution for 20 min after the methanol had been removed. With a 100× magnification, an inverted microscope was used to examine the dyed biofilms. Instead, after staining, the excess dye was removed with tap water, and the dye present in the cells of the biofilm that had grown on the well’s walls was then soluble in a solution of 33% acetic acid. In order to understand the data based on the spectrophotometric reading of the colored suspension’s absorbance at 490 nm, suspensions were prepared in this manner.

## 3. Results

### 3.1. X-ray Diffraction

**Figure 1:**
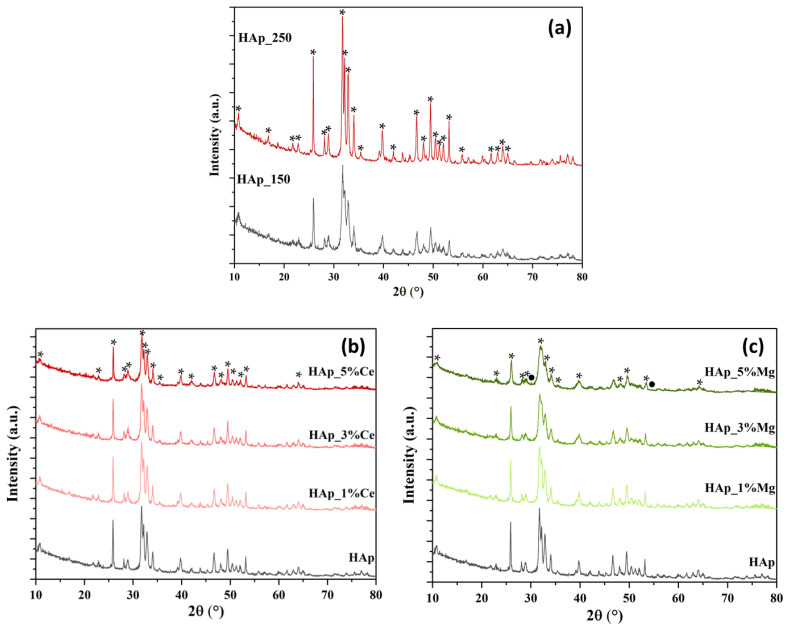
X-ray diffractogram for hydroxyapatite powders synthesized at different parameters of microwave-assisted hydrothermal treatment (**a**) and hydroxyapatite powders substituted with cerium (**b**) and magnesium (**c**) ions at concentrations of 1, 3, and 5% (*—HAp, ●—Whitlockite).

Figure 1a shows the X-ray diffractograms for the hydroxyapatite powders synthesized using different microwave-assisted hydrothermal treatment parameters. As can be seen, the only crystalline phase identified is hydroxyapatite in the hexagonal crystallization system P63/m, according to ICSD file 01-073-8419. However, increasing the temperature leads to an increase in the crystallinity of the samples, shown by the high intensity of the corresponding diffraction peaks. Figure 1b shows the X-ray diffractograms for cerium ion-substituted hydroxyapatite powders at 1, 3, and 5% concentrations. As can be seen, boosting the concentration of the substituent leads to a decrease in the intensity of the diffraction peaks and, implicitly, in the crystallinity of the sample. However, the only crystalline phase identified is hydroxyapatite, which confirms the success of the substitution reaction by the absence of the formation of other phases, such as cerium oxide or cerium phosphate. In the case of substitution with magnesium ions at concentrations of 1, 3, and 5%, the diffractograms show different behavior compared to the case of substitution with cerium (Figure 1c, Table 1). On the one hand, 1 and 3% concentrations do not produce considerable differences in the powders’ crystallinity but reduce the crystallite’s average size. However, in the case of the 5% substituted hydroxyapatite sample, the appearance of new diffraction maxima attributed to the magnesium whitlockite crystalline phase, a mixed calcium and magnesium phosphate with the chemical formula Ca_9_Mg(HPO_4_)(PO_4_)_6_ (ICSD 04-008)-8604). In this case, the average crystallite size attributed to hydroxyapatite decreases insignificantly; the expansion in the degree of crystallinity is associated with the large crystallite size related to whitlockite. Despite the development of a new phase, the applicability of magnesium ion-substituted hydroxyapatite powder at a concentration of 5% in the medical field is justified by the abundance of this phase in natural bone [54,55].

The results obtained from Rietveld refinement (Table 1) confirm the increase in crystallinity of the sample with increasing temperature. Moreover, the high crystallinity of the sample treated at the temperature of 250 °C is due to the increase in crystallite size, a natural process encountered in this type of synthesis. Specifically, with the increase in pressure, temperature, or reaction time, the size of the crystallites and, implicitly, of the nanoparticles increases proportionally. The results of the Rietveld refinement confirm the previous observations, namely the reduction of the crystallinity of the powders concomitant with the decrease of the crystallite size, are presented in Table 1.

### 3.2. Dynamic Light Scattering (DLS) and Zeta Potential

**Figure 2:**
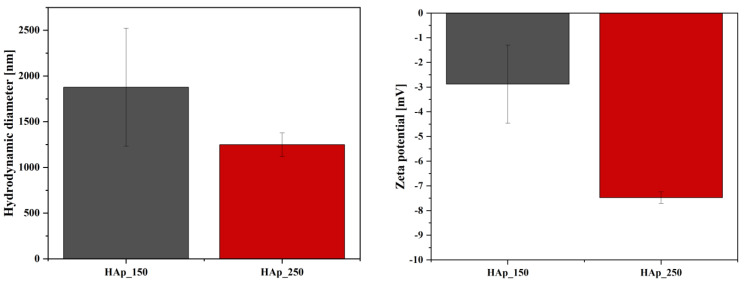
Hydrodynamic diameter and zeta potential values for hydroxyapatite treated at 150 and 250 °C.

Figure 2 shows the hydrodynamic diameter values of hydroxyapatite nanoparticles treated at two distinct temperatures (150 °C and 250 °C). In this context, the hydrodynamic diameter is lower for the sample treated at 250 °C, which may be due to larger particle size and, consequently, a reduced surface reactivity that would lead to particle agglomeration. The results are confirmed by the zeta potential values (Figure 2), where more negative values are recorded for this sample, so increased stability prevents particle aggregation. Additionally, the results follow the hypothesis behind the hydrothermal treatment of the nanoparticles, as higher temperatures are expected to generate particle growth. However, the differences between the two samples in terms of stability are not significant.

**Figure 3:**
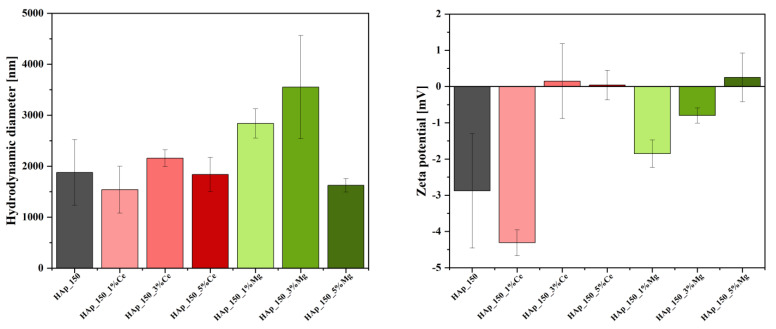
Hydrodynamic diameter and zeta potential values for hydroxyapatite treated at 150 °C and substituted with cerium and magnesium ions at concentrations of 1, 3, and 5%.

Figure 3 shows the hydrodynamic diameter values of hydroxyapatite nanoparticles substituted with cerium and magnesium ions at concentrations of 1, 3, and 5%. Thus, the high degree of agglomeration can be observed in the case of all the samples, a fact also confirmed by the low values of the zeta potential (Figure 3). Furthermore, in both types of substitutions, increasing concentrations lead to the increase of the zeta potential values, i.e., from negative values to positive values. However, the substitution with cerium ions at the concentration of 1% seems to increase the stability of the hydroxyapatite nanoparticles, also shown by the hydrodynamic diameter values. Further increasing the substitution concentration leads to a decrease in nanoparticle stability, as the zeta potential values are close to 0 mV with no significant differences between the 3% and 5% concentrations, and in nanoparticle size, as compared to the HAp_150 sample. However, the presence of whitlockite at the level of the hydroxyapatite sample substituted with 5% magnesium leads to a considerable decrease in the hydrodynamic diameter compared to the samples substituted with 1 and 3%, which also denotes a growth in particle size, therefore a decrease in surface reactivity, with the formation of this phase. This could be explained by the defects occurring in the unit cell of hydroxyapatite due to the introduction of magnesium ions.

### 3.3. Scanning Electron Microscopy (SEM)

**Figure 4:**
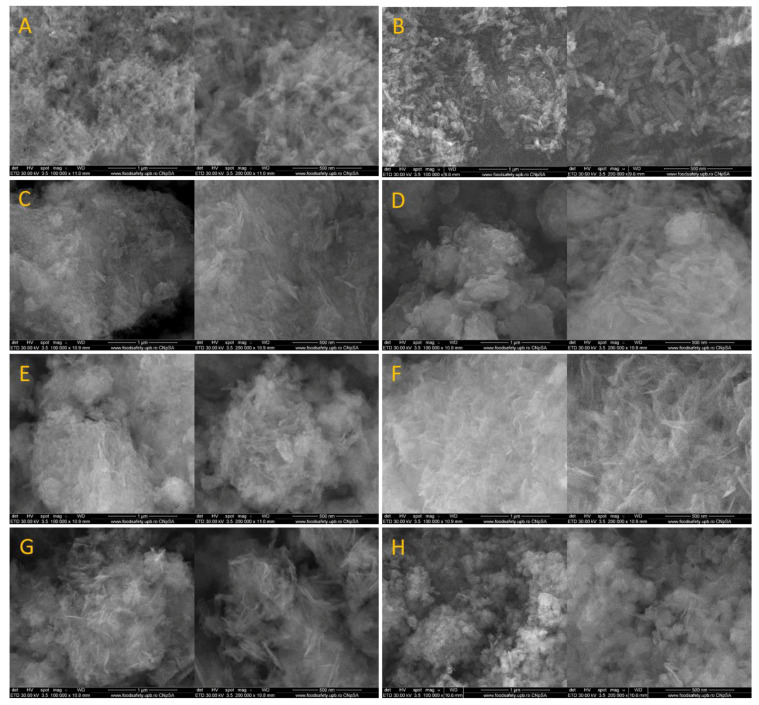
SEM micrographs recorded for HAp_150 (**A**), HAp_250 (**B**), HAp_Ce_1% (**C**), HAp_Ce_3% (**D**), HAp_Ce_5% (**E**), HAp_Mg_1% (**F**), HAp_Mg_3% (**G**), HAp_Mg_5% (**H**).

**Figure 5:**
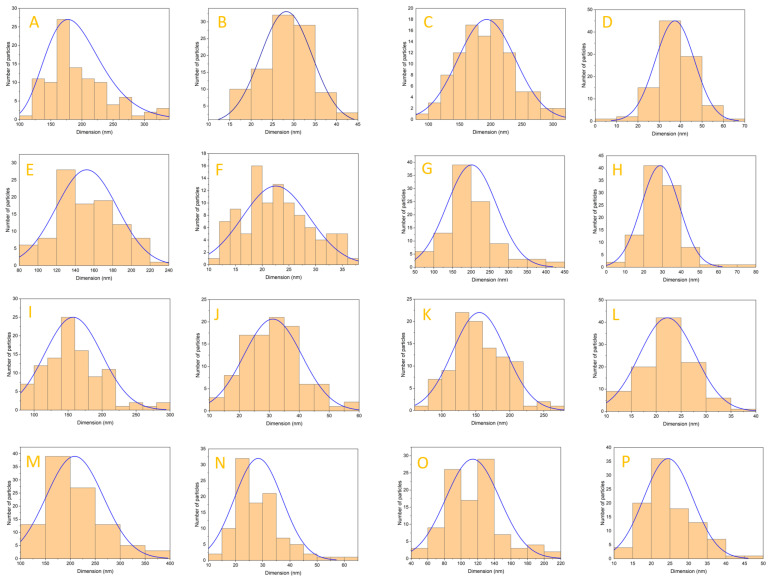
Dimensional distribution for HAp_150, HAp_250, HAp_Ce_1%, HAp_Ce_3%, HAp_Ce_5%, HAp_Mg_1%, HAp_Mg_3%, HAp_Mg_5% by length (**A**,**C**,**E**,**G**,**I**,**K**,**M**,**O**) and by diameter (**B**,**D**,**F**,**H**,**J**,**L**,**N**,**P**).

Figure 4A shows the SEM micrographs obtained for the unsubstituted HAp powder obtained at 150 °C and the dimensional distribution of the particles (Figure 5A,B). In this case, the micrographs obtained show the rod-like morphology characteristic of hydroxyapatite, similar to the physiological one found in the structure of hard tissue. From a dimensional point of view, this falls into the field of nanomaterials, the rods having a diameter between 15 and 45 nm, the average size being 28.23 nm. The morphology of the hydroxyapatite powder is preserved with the increase of the heat treatment temperature; thus, in Figure 4B, better-contoured rods can be observed than in the previous case, with slightly increased dimensions for the HAp_250 sample. Thus, an increase in the average diameter to 37.29 nm (Figure 5D) is observed for the hydroxyapatite treated at 250 °C, with the increase in temperature. Regarding the length distribution, in this case, values between 80 and 320 nm can be observed, with an average size of 190 nm (Figure 5C). This dimensional increase is in good correlation with the X-ray diffraction results regarding the crystallite size.

Following the morphological evaluation, it is observed that the cerium substitution does not substantially change the appearance of the hydroxyapatite. Thus, all three concentrations (1%, 3%, 5%) maintain the rod-like structure with nanometric dimensions found in the case of unsubstituted powders. In this sense, their large specific surface area may conduct the formation of agglomerates that can be observed, especially in the case of the three samples of hydroxyapatite substituted with cerium (Figure 4C–E). Analyzing the histograms made for the three cerium-doped hydroxyapatite samples (Figure 5E–J), it can be seen that the diameter of the rods is between 10 and 80 nm and increases with increasing cerium concentration. Considering the distribution by length, a dimensional inhomogeneity can be observed, with values between 50 and 450 nm.

The magnesium-substituted hydroxyapatite samples were also subjected to SEM analysis, the results being shown in Figure 4F–H. The three magnesium concentrations (1%, 3%, 5%) influence the hydroxyapatite morphology compared to the cerium-substituted samples. In the case of the HAp_Mg_1% sample, the rod-type architecture is preserved, but with the increase in magnesium concentration, small irregular platelets begin to appear (average diameter of 114.07 nm and average thickness of 24.54 nm), interconnected with the characteristic rods of hydroxyapatite. The sample with the highest concentration of magnesium (5%) shows the highest proportion of such platelets and a very low presence of rods.

Also, all three samples present dimensional characteristics within the nanometric range, with a high tendency to agglomerate. It can be seen for the samples with magnesium concentrations of 1% and 3% that both the diameter and the length increased with increasing concentration.

**Figure 6:**
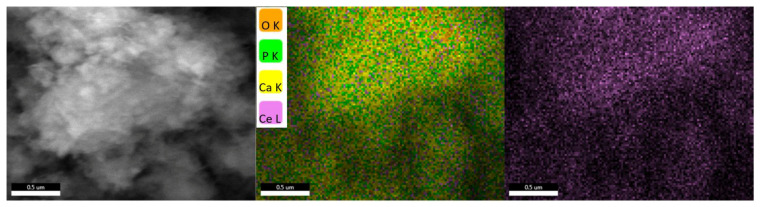
Elemental mapping recorded for HAp_Ce_5%.

Analyzing the hydroxyapatite powder with the highest proportion of cerium, the presence of the substituent element is observed, uniformly distributed alongside the element’s characteristic of hydroxyapatite (Figure 6).

**Figure 7:**
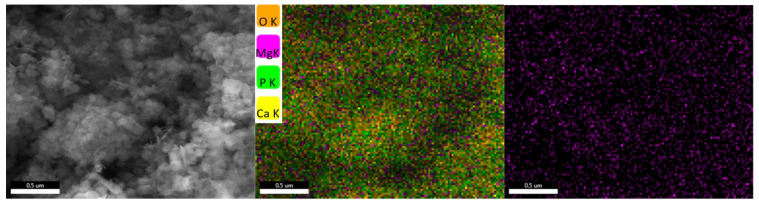
Elemental mapping recorded for HAp_Mg_5%.

A similar behavior is also observed analyzing the hydroxyapatite powder with the highest proportion of magnesium, where the presence of the substituent element uniformly distributed alongside the elements characteristic of hydroxyapatite is observed (Figure 7).

### 3.4. Fourier-Transform Infrared Spectroscopy (FT-IR)

**Figure 8:**
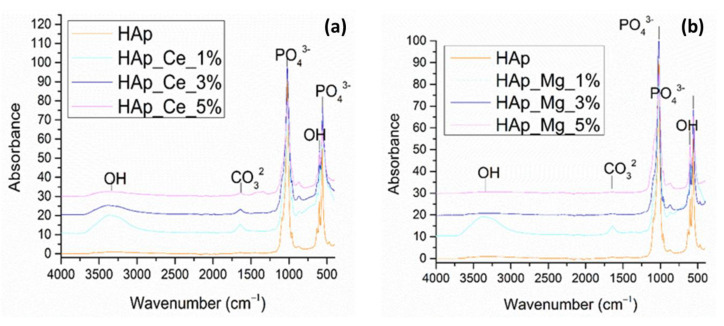
FT-IR spectra recorded for HAp_150, HAp_Ce_1%, HAp_Ce_3%, HAp_Ce_5% (**a**) and Hap_150, HAp_Mg_1%, HAp_Mg_3%, HAp_Mg_5% (**b**).

The FT-IR spectra of HAp_Ce (1%, 3%, 5%) and HAp_150 powders show bands corresponding to the vibrational modes of the different functional groups that were found in the sample. Thus, the cerium-substituted hydroxyapatite samples are similar in terms of molecular structure to the plain hydroxyapatite sample. However, a difference is observed by the presence of the O–H group at the wave number of about 3380 cm^−1^ found in the cerium samples, attributed to water molecules adsorbed on the surface. The vibrational band at wave number 1022 cm^−1^ is characteristic of the PO_4_^3−^ group, characteristic of hydroxyapatite. Also, in the case of samples substituted with cerium, the debut of the band at approximately 1638 cm^−1^ confirms the carbonation of the samples, being attributed to the CO_3_^2−^ group, a process that occurs when the powders come into contact with the atmosphere (Figure 8a).

Also, the FT-IR results obtained for the HAp_150, and HAp_Mg (1%, 3%, 5%) samples, show the similarity between the spectra for magnesium-substituted hydroxyapatite and simple hydroxyapatite can be observed. Its carbonated nature is also observed compared to the other samples. At the wave number from approximately 1022 cm^−1^, the imprint of hydroxyapatite is observed through the PO_4_^3-^ group (Figure 8b).

### 3.5. Biological Evaluation of HAp Samples

**Figure 9:**
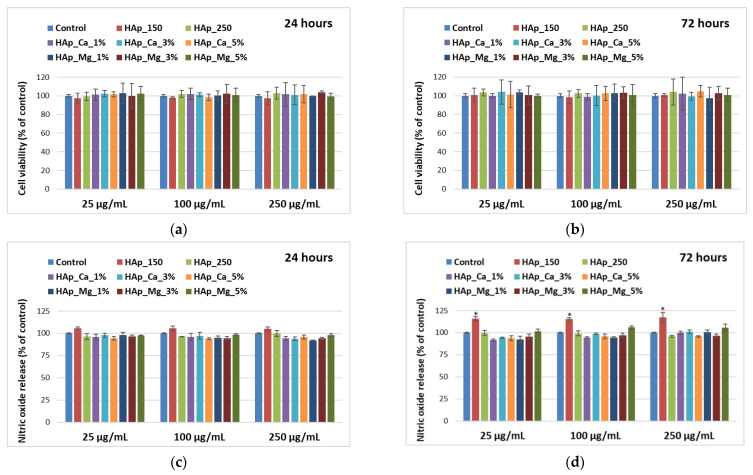
Viability level (**a**,**b**) and nitric oxide release (**c**,**d**) of MC3T3-E1 osteoblasts cultured in the presence of different concentrations (25, 100, and 250 µg/mL) of HAp samples for 24 h and 72 h. Results are calculated as means ± SD (*n* = 3) and represented relative to control (cells that were not incubated with HAp samples); the statistical analysis: two-tailed Student’s test * *p* ≤ 0.05 were considered as statistically significant.

MC3T3-E1 osteoblast cells were used to analyze the biological behavior of hydroxyapatite samples with cerium and magnesium ions by both quantitative (MTT viability and NO cytotoxicity assays) and qualitative (fluorescence microscopy) evaluation. The metabolic activity of osteoblasts incubated with HAp samples was evaluated and was comparable with the levels obtained for control as revealed by MTT assay (Figure 9a,b), proving that all types of tested samples can represent biocompatible substrates for normal osteoblastic cell proliferation. Also, no statistically significant changes were observed compared to the control for both time intervals (24 h and 72 h), confirming the absence of cytotoxicity of HAp-based suspensions after three days of incubation. Furthermore, the levels of NO release after the HAp exposure were not significantly different compared to the control (Figure 9c,d), confirming the good biocompatibility in the presence of these samples. However, a slight increase of 15% above the control level was noticed in the case of HAp_150 after 72 h. This change could be explained by its higher crystallite size (46.13 nm) compared to the other samples, which have a size lower than 20 nm. This characteristic could influence the release of different intracellular molecules, including the NO, to the culture medium, by affecting cell permeability. 

**Figure 10:**
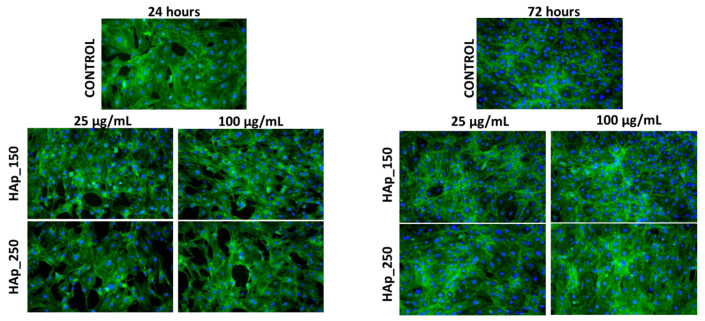
Fluorescence micrographs of MC3T3-E1 osteoblasts cultured for 24 h and 72 h in the presence of different concentrations (25 and 100 µg/mL) of HAp_150 and HAp_250 samples. The actin cytoskeleton is stained with phalloidin-FITC (green) and nuclei with DAPI (blue). All images were captured with a 10× objective.

**Figure 11:**
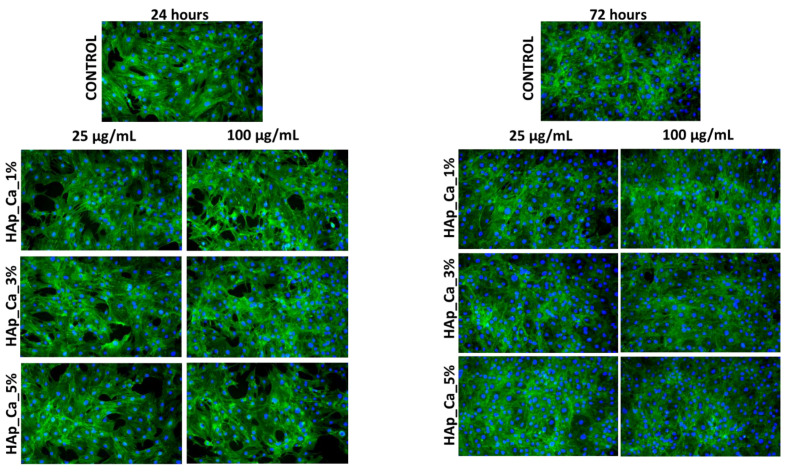
Fluorescence micrographs of MC3T3-E1 osteoblasts cultured for 24 h and 72 h in the presence of different concentrations (25 and 100 µg/mL) of HAp_Ce_1%, HAp_Ce_3%, HAp_Ce_5% samples. The actin cytoskeleton is stained with phalloidin-FITC (green) and nuclei with DAPI (blue). All images were captured with a 10× objective.

**Figure 12:**
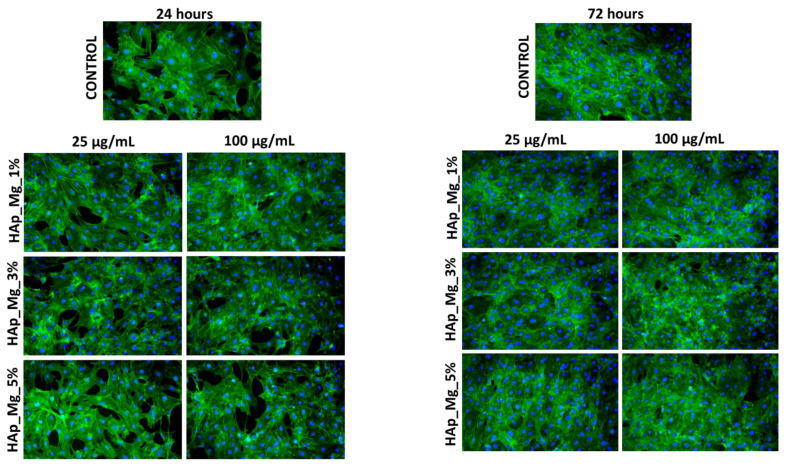
Fluorescence micrographs of MC3T3-E1 osteoblasts cultured for 24 h and 72 h in the presence of different concentrations (25 and 100 µg/mL) of HAp_Mg_1%, HAp_Mg_3%, HAp_Mg_5% samples. The Actin cytoskeleton is stained with phalloidin-FITC (green) and nuclei with DAPI (blue). All images were captured with a 10× objective.

Fluorescence microscopy experiments, which allowed the cytoskeleton and nuclei visualization, have offered additional details on the biological effects of HAp-based samples. The fluorescent micrographs in Figure 10, Figure 11 and Figure 12 demonstrate that MC3T3 cells had uniform spreading and good adherence after being cultured for 24 and 72 h with the investigated substances. The cells also had a typical osteoblast-like phenotype and normal cell shape. (flattened structure, multiple cytoskeleton extensions, elongated actin filaments, and prominent central nuclei).

### 3.6. Microbiological Evaluation

#### 3.6.1. MIC (Minimum Inhibitory Concentration) Method

**Figure 13:**
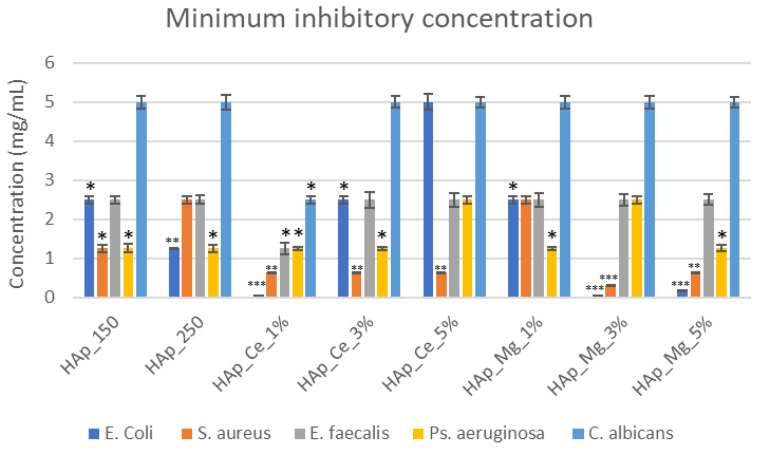
Minimum Inhibitory Concentration for hydroxyapatite samples. Results are calculated as means ± SD (n = 3); the statistical analysis: two-tailed Student’s test * *p* ≤ 0.05, ** *p* ≤ 0.01, *** *p* ≤ 0.001 were considered as statistically significant (for each strain).

The minimum inhibitory concentration was evaluated after 24h. The concentration where the MIC was observed is presented in Figure 13. As observed, the MIC values range from 0.1–2.5 mg/mL for bacteria strains and 5 mg/mL for *C. albicans*.

#### 3.6.2. Development of Monospecific Biofilms

**Figure 14:**
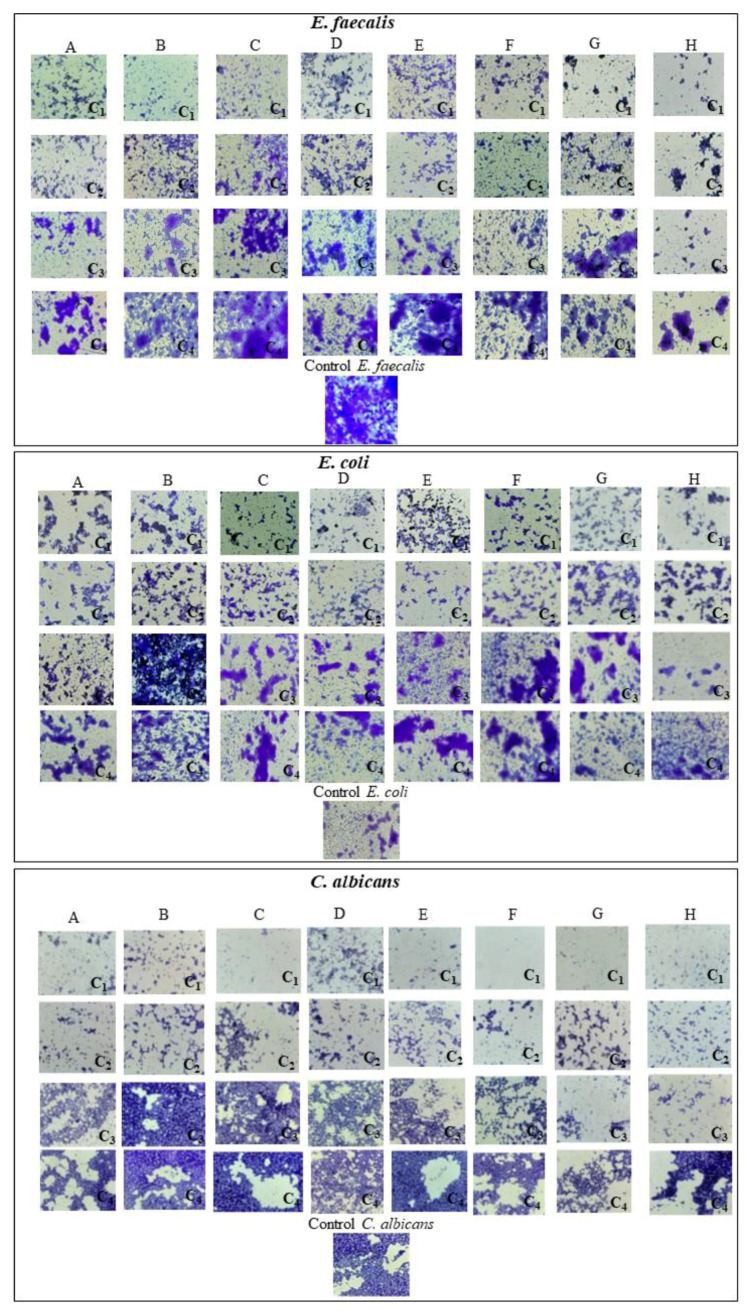
Optical microscopy for hydroxyapatite samples for *E. faecalis*, *E. coli*, *C. albicans*. A-HAp_150, B-HAp_250, C-HAp_Mg_1%, D-HAp_Mg_3%, E-HAp_Mg_5%, F-HAp_Ce_1%, G-HAp_Ce_3%, H-HAp_Ce_5%, C1-Concentration 1 (5 mg/mL), C2-Concentration 2 (2.5 mg/mL), C3-Concentration 3 (0.62 mg/mL), C4-Concentration 4 (0.15 mg/mL).

After 24 h, the developed monospecific biofilms were evaluated by optic microscopy (Figure 14)*;* it was observed that the hydroxyapatite substituted with cerium inhibited bacteria better than samples unsubstituted and hydroxyapatite substituted with magnesium. Figure 14 below reveals that the biofilms develop differently in the presence of HAp powders, depending on their concentrations; usually, concentrations above 2.5 mg/mL reveal a significant inhibition, as shown in the microscopy images. 

**Figure 15:**
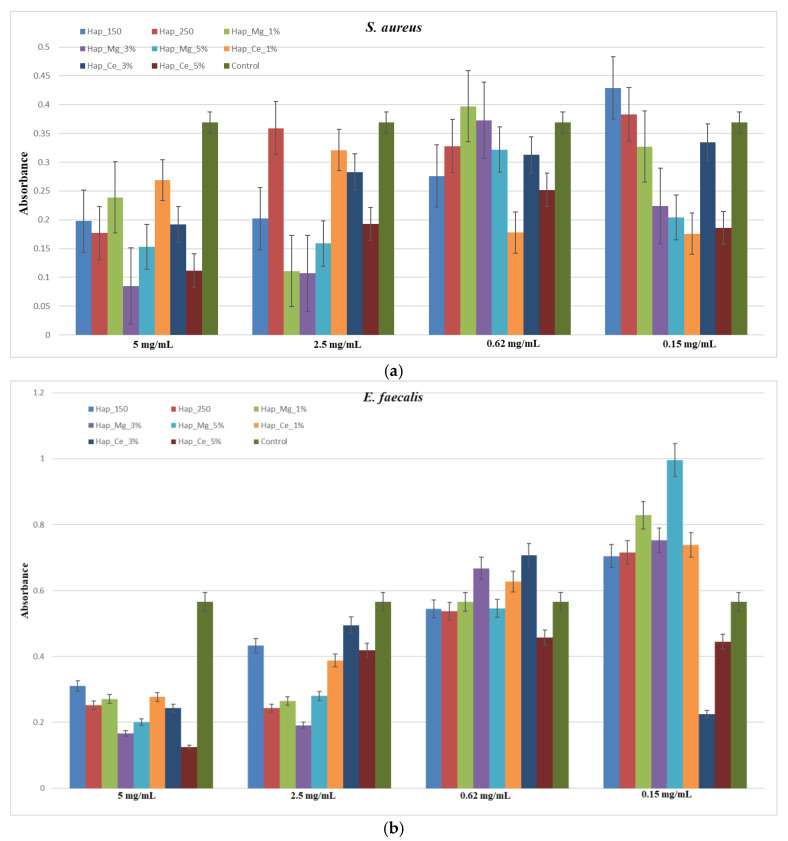

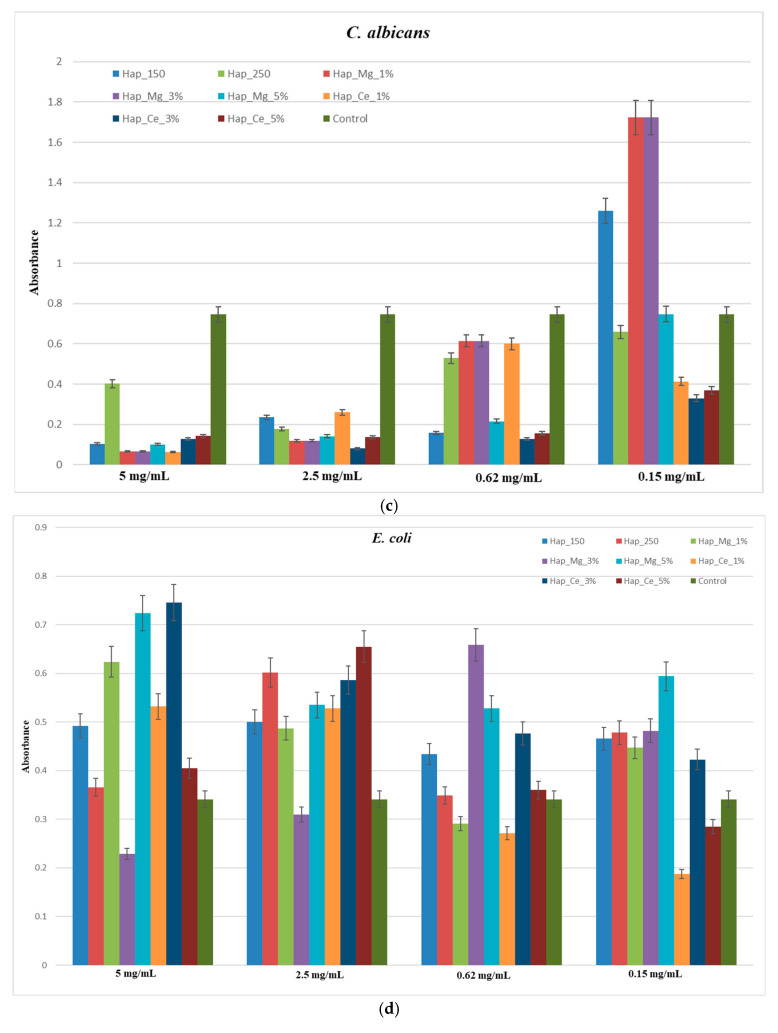

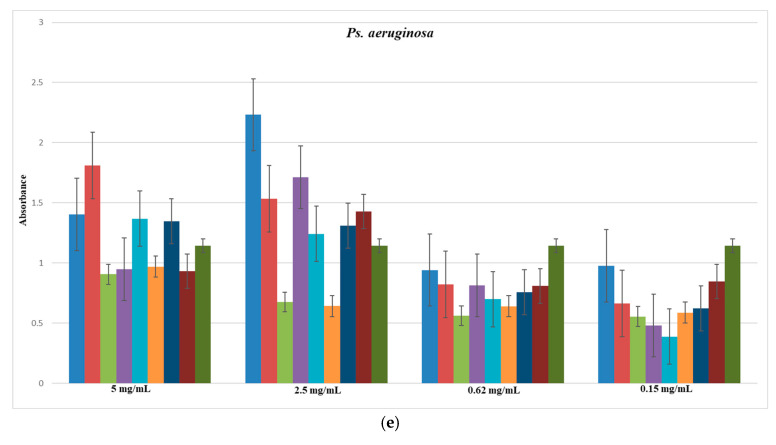
Development of monospecific biofilm of (**a**) *S. aureus*, (**b**) *E. faecalis*, (**c**) *C. albicans*, (**d**) *E. coli*, (**e**) *Ps. aeruginosa*.

OD 492nm (Abs) results confirmed that hydroxyapatite samples could interfere with *E. faecalis*, *E. coli.*, *S. aureus*, *P. aeruginosa*, and *C. albicans* biofilm formation. (Figure 15a–c). The hydroxyapatite substituted with cerium showed improved bacteria inhibition, as compared with hydroxyapatite substituted with magnesium. For the Gram-negative bacteria such as *E. coli* and *P. aeruginosa*, the samples showed a limited effect (Figure 15d,e).

## 4. Discussion

### 4.1. X-ray Diffraction

HAp-based biomaterials can be easily designed using the microwave-assisted hydrothermal process since modifying different nanoparticle features, such as dimension, is simple. Additionally, this adaptable method can increase the bioactivity and biofunctionality of HAp by substituting it with different inorganic ions [56]. Figure 1 depicts the phases of pure hydroxyapatite (HAp), Ce(III)-substituted hydroxyapatite, and Mg-substituted hydroxyapatite formed by microwave-assisted hydrothermal, respectively. The calcium phosphate in Figure 1’s XRD image can be seen as a single phase with all relevant diffraction peaks. For cerium-substituted samples, a recent study showed that compared to pure hydroxyapatite, all Ce(III)-substituted hydroxyapatite samples may exhibit a drop in X-ray peak intensities with increasing ion doping concentration levels [57]. In our situation, a similar decrease in diffraction peak strength was seen when the cerium concentration increased. However, the sole crystalline phase found is hydroxyapatite, indicating that the substitution reaction was successful because no additional phases, such as cerium oxide or cerium phosphate, were formed. In the case of substitution with magnesium ions at concentrations of 1, 3, and 5%, the diffractograms show different behavior compared to substituting with cerium (Figure 1c, Table 1). On the one hand, 1 and 3% concentrations decrease the average size of the crystallites while making little to no effect on the powders’ crystallinity. The occurrence of new diffraction maxima in the 5% magnesium substituted hydroxyapatite sample was attributed to the mixed calcium and magnesium phosphate crystal phase known as magnesium whitlockite. Additionally, Table 1 shows that for both cerium and magnesium, the crystallite size decreases as concentration increases. The main difference appears for the sample with 5% magnesium; the crystallite size has been increased due to the formation of whitlockite. Additionally, it can be seen that the crystallite size increases due to the higher temperature for the HAp produced at 250 °C. Whitlockite, or magnesium whitlockite, is a calcium orthophosphate crystal in which calcium and magnesium are partially substituted under biological conditions. The difference is observed in X-ray or electron diffraction patterns and occurs in extra- or intra-tissular places under normal or pathological settings, primarily in tissues of non-epithelial origin [58,59]. By using X-ray diffraction, a recent study examined a variety of pathological calcifications and found that whitlockite and apatite appeared to be present often [60].

### 4.2. Dynamic Light Scattering (DLS) and Zeta Potential

Through measurements of the hydrodynamic diameter and zeta potential, the stability of pure HAp, Ce(III)-substituted hydroxyapatite, and Mg-substituted HAp was evaluated. The Rayleigh scattering from suspended nanoparticles moving with Brownian motion is the foundation of the dynamic light scattering (DLS) method. By shining a laser source on the sample, we can observe the particle diffusion velocity and calculate the hydrodynamic diameter of the nanoparticles [61].

From the DLS analysis, it was concluded that the hydrodynamic diameter is lower for the sample synthesized at 250 °C temperature because of larger particle size and reduced surface reactivity; the zeta potential also confirmed the increased stability for this sample.

For the samples of hydroxyapatite substituted with cerium and magnesium, it can be observed a high degree of agglomeration, while also having a low zeta potential. The most important difference is observed in the hydroxyapatite sample substituted with 5% magnesium which has a decrease in the hydrodynamic diameter, showing an increase in particle size.

### 4.3. Scanning Electron Microscopy (SEM)

From the SEM micrographs in Figure 4, it can be seen that the HAp powder sample has a rod-shaped form with width and length comparable to those of hard tissue (tens and hundreds of nanometers, respectively). It can be shown that the nanoparticles, in the case of HAp_250, have larger diameters, with the dimension expanding as the heat temperature rises. The X-ray diffraction results regarding the crystallite size are well correlated with this dimensional increase. Figure 5A,B depicts the dimensions distribution of the particles, while Figure 4A displays the SEM micrographs for the unsubstituted HAp powder obtained at 150 °C. The acquired micrographs, in this instance, exhibit the rod-like morphology that characterizes hydroxyapatite and is comparable to the physiological one observed in the composition of hard tissue. This is a type of nanomaterial from a dimensional perspective, with rods that range in diameter from 15 to 45 nm and have an average size of 28.23 nm. Because the morphology of the hydroxyapatite powder is conserved while the heat treatment temperature is raised, better-contoured rods with slightly larger diameters for the HAp_250 sample can be seen in Figure 4B. As a result, for the hydroxyapatite treated at 250 °C, an increase in the temperature causes the average diameter to grow to 37.29 nm (Figure 5D). In this instance, values between 80 and 320 nm with an average size of 190 nm can be seen in the length distribution (Figure 5C). The results of the X-ray diffraction regarding the crystallite size are well correlated with this dimensional increase. Following morphological analysis, it is found that the cerium substitution has little effect on the hydroxyapatite’s appearance. As a result, the rod-like structure with nanometric dimensions present in the case of unsubstituted powders is maintained at all three concentrations (1%, 3%, and 5%). In this way, their huge specific surface area causes the development of observable agglomerates. Similar findings were recently published in a study using hydroxyapatite doped with Ce(III) and Ce(IV), where it was revealed that the dimension varies depending on the concentration [57,62]. The findings of the SEM examination performed on the magnesium-substituted hydroxyapatite samples are depicted in Figure 4F–H). Comparatively to the cerium-substituted samples, the three magnesium concentrations (1%, 3%, and 5%) impacted the hydroxyapatite morphology. The rod-type architecture is still present in the HAp_Mg_1% sample, but as the magnesium concentration rises, little irregular platelets with an average diameter of 114.07 nm and an average thickness of 24.54 nm start to emerge being interconnected with the characteristic rods of hydroxyapatite. The sample with the highest magnesium content (5%) exhibits the largest percentage of these platelets and the least number of rods. Whitlockite synthesis was explained by Macha et al. [63], and the addition of magnesium to hydroxyapatite was observed to alter particle size in a study presented by Nigar et al., intriguingly, at a higher temperature (i.e., 350 °C) in this system, a tubular morphology of HA and rhombohedral-shaped Mg-WH particles were seen [64].

### 4.4. Fourier-Transform Infrared Spectroscopy (FT-IR)

When the hydroxyapatite powders with the largest amounts of cerium and magnesium are analyzed, the substituent elements are present and consistently distributed with the hydroxyapatite-specific elements.

Unsubstituted hydroxyapatite (HAp_150 and HAp_250) IR spectra exhibit absorption maxima that are unique to hydroxyapatite, including stretching vibrations from structural hydroxyl groups (~3530 cm^−1^), ν3 asymmetric stretching of the ${\mathrm{PO}}_{4}^{3-}$(~1110, ~1080, and ~1050 cm^−1^) and ${\mathrm{PO}}_{4}^{3-}$ ν1 stretching (~990 and ~880 cm^−1^) [65,66]. The overlapping stretch of OH (hydroxyl groups) bound in HAp may be the cause of the weaker peak at ~3500 cm^−1^. The presence of the O-H group at about 3380 cm^−1^ in the cerium samples is attributed to water molecules adsorbed on the surface, distinguishing the hydroxyapatite substituted with cerium. The emergence of the band at roughly 1638 cm^−1^, which is attributed to the ${\mathrm{CO}}_{3}^{2-}$ group and indicates the carbonation of the samples in both the cerium-substituted samples as well, is a process that takes place when the powders come into contact with the atmosphere. The decrease in HAp crystallinity is related to the increase of Ce^3+^ ion concentration, which reduced band intensity [67]. The XRD study provided additional support for these conclusions. In comparison to the other samples, its carbonated character is also observed. The imprint of hydroxyapatite is visible through the ${\mathrm{PO}}_{4}^{3-}$ group at a wave number of about 1022 cm^−1^ [68]. In an investigation based on the investigation of magnesium incorporated in hydroxyapatite, a peak at 3698 cm^−1^ was found to match the stretching mode of hydroxyl groups that manifests when associated with magnesium, showing the existence of Mg^2+^ in the apatite structure [69].

### 4.5. Biological Behavior Evaluation on Osteoblast Cells

Due to its osteoconductivity and osteoinductivity, hydroxyapatite (HA), which has a composition and structure highly very much alike to those of natural bone minerals, has been regarded as the best material to construct bone tissue engineering scaffolds [70]. According to research by Vieria et al. on the effects of hydroxyapatite-containing cerium on fibroblast, none of the tested samples had viability levels consistent with a cytotoxic effect [40]. A study using MG-63 osteoblast cell lines obtained from human osteosarcoma provided similar results as well [71]. MC3T3 cells were used to perform the MTT assay on the samples obtained in our study. The samples were evaluated at 24 and 72 h, and it was found that at neither time point did they have any harmful effects on the cells supporting the normal development and proliferation of osteoblastic cells, the metabolic activity being comparable with control. Also, the samples did not produce NO release; their level was kept close to the control values for all tested samples. In the instance of the magnesium-substituted hydroxyapatite and cerium-substituted hydroxyapatite, the metabolic activity of each HAp powder was compared with the control, and the MTT assay (Figure 9a,b) demonstrated that all recommended samples are appropriate substrates for normal osteoblastic cell growth and proliferation. MC3T3-E1 cells showed good adherence and homogeneous spreading onto the substrate when treated with HAp-based coatings for 24 h. The cells also had a typical osteoblast-like phenotype and normal cell shape according to fluorescence micrographs (Figure 10, Figure 11 and Figure 12).

A recent study observed that, according to the hypothesized osteoprotective action of Mg, certain proteins involved in osteogenesis were much more regulated in the presence of Mg discs than in Ti-disc. Due to its propensity for biodegradation and outstanding biocompatibility, magnesium is a promising metal for biodegradable implant applications. Compared to Ti metal discs, the overall effect of decomposing Mg on osteoblast is noticeably bigger and more complex [72,73,74]. In the case of cerium, recent research has shown that hydroxyapatite substituted with cerium and Fe_3_O_4_ may become toxic due to Ce and Fe release at higher concentrations [75]. In our study, the samples with all three cerium concentrations (1%, 3%, 5%) did not show any toxic effect on the cells. In a recent study based on hydroxyapatite substituted with cerium, cellular viability was also maintained for a concentration of 10% cerium [57].

### 4.6. Microbiological Evaluation

In order to create implants that are more resistant to bacterial colonization, advances have been made in the field of implant surface engineering, with implant-associated bone and joint infection (BJI) being a rare but devastating side effect of arthroplasty and orthopedic trauma. The number of implant-associated infections is rising due to the expanding number of implant devices, despite technical and medical efforts to prevent such illnesses [76].

The development of antimicrobial protection against the adhesion and growth of microbial biofilms was one of the anticipated uses of the created bioactive coatings. After forming on the surface of a prosthetic device or implant, biofilms are exceedingly difficult, if not impossible, to treat due to their strong resistance to antibiotics and host immunity; the damaged implant is typically removed as a result [77]. As a result, it is still difficult for materials science and many medical disciplines to prevent biofilm growth on the implant surface. 

The development of new materials with enhanced resistance to microbial colonization or bioactive implant coatings capable of delivering an antimicrobial effect close to the implanted material by first encapsulating and then locally releasing conventional antibiotics or other antimicrobial agents are two research directions that are currently being pursued [78,79,80,81]. Along with their inherent bioactivity, HAp-based coatings have a remarkable ability for immobilizing or encasing antimicrobial agents. Inorganic structures, including bismuth, cerium, copper, magnesium ions, silver ions, and nanoparticles, and zinc ions and nanoparticles have been found to have enhanced anti-pathogenic properties [82]. 

In the case of hydroxyapatite substituted with cerium, recently, it was reported in a study that after a 24 h incubation period, the cerium substituted hydroxyapatite’s antibacterial activity against the pathogens *E. coli* 714 and *S. aureus* ATCC 6538 was investigated. The antibacterial activity of pure hydroxyapatite (xCe = 0), which served as the standard, is compared to the antimicrobial activity of cerium-substituted hydroxyapatite. A prior study found that pure HAp had no bactericidal activity against *E. coli* 714 and *S. aureus* ATCC 6538 [83]. According to the study results, *E. coli* 714 bacterial strain survival rates decline as Ce concentrations in hydroxyapatite rise. When the amount of Ce in the hydroxyapatite increases, the S. aureus ATCC 6538 bacterial strain’s survival rate declines [84]. Recently, it has been proposed that *E. faecalis*’ capacity to develop biofilms plays a significant role in the pathophysiology of enterococci infections [85]. *E. faecalis* forms a multilayer, antibiotic-resistant biofilm by secreting a protective extracellular matrix that adheres to both biotic and abiotic surfaces. This intrinsic antibiotic resistance represents an important challenge to treating enterococci infections [86]. In accordance with a recent study based on hydroxyapatite substituted with rare earth elements, both HAp and Nd-Ce/HAp exhibited an inhibitory zone against all of the bacteria test species. The synthesized hydroxyapatite without substitution and HAp with neodymium and cerium displayed antibacterial and antifungal activities. Regarding *S. aureus*, *S. mutans*, and *S. epidermidis*, the inhibitory zone was nearly within reach for both HAp and Nd-Ce/HAp. Nd-Ce/HAp has a larger inhibition zone for *E. coli* than HAp, which showed a larger inhibition zone for fungi. Commercial HAp did not affect Gram-positive or Gram-negative bacteria. Surprisingly, the method used to synthesize hydroxyapatite has a significant influence on how effective it is against bacteria. For example, HAp samples made using the microwave-assisted combustion method demonstrated superior resistance to both Gram-positive and Gram-negative bacteria [73].

The antibacterial qualities of magnesium have been connected to several mechanisms that are related to those shown in the case of other metallic ions. According to the literature, nanoparticles harm human organisms through indirect DNA damage, oxidative stress, and inflammation. Additionally, the generation of reactive oxygen species (ROS), which may conduct oxidative DNA damage, protein denaturation, and lipid peroxidation, has been linked to the toxicity of metal oxide nanoparticles. Reactive oxygen species (ROS) generation has also been linked to the toxicity of magnesium oxide nanoparticles, as it has been for other metal oxide nanoparticles. It has also been demonstrated that MgO nanoparticles produce large quantities of magnesium ions; however, these ions do not exhibit appreciable toxicity. The harmful effects of MgONPs have been studied by many researchers utilizing fish as model organisms [86,87,88,89,90].

Studies on the effects of magnesium addition to hydroxyapatite highlighted that MgHAp suspensions, compared to both the control and HAp suspensions, demonstrated good antibacterial activities even after 24 h in the case of Gram-negative *P. aeruginosa* and Gram-positive *S. aureus* bacterial strains. The results of the antimicrobial assays showed that HAp suspensions tended to make it easier for all of the microbial cells under investigation to form biofilms at all of the examined concentrations and time intervals. These findings show that when magnesium is combined with hydroxyapatite, it gives the MgHAp solutions their antibacterial properties. A recent study showed that *P. aeruginosa’s* ability to produce biofilms was also decreased by the MgHAp solution at all concentrations tested, covering from 5 to 0.009 mg/mL; however, for the *S. aureus* bacterial strain, the inhibition was first noted at 2.5 mg/mL. In contrast, *P. aeruginosa* and *S. aureus* bacterial strains were more likely to produce biofilms when exposed to the HAp solutions than when not exposed, even after 24 h of incubation. For doses ranging from 5 to 1.25 mg/mL of the MgHAp suspensions, a mild suppression of biofilm development was also seen after 24 h in the instance of the fungus *C. albicans*. More than that, HAp suspensions had a stimulating effect on the *E. faecalis* and *E. coli* biofilm development [89].

In a further study, the antibacterial effectiveness of the HAp/MgO spherical granules was assessed in terms of planktonic development and early bacterial adherence against three of the most significant bacteria reported in orthopedic illnesses. It was demonstrated that HAp/MgO composites sintered at 900 and 1100 °C were able to significantly limit bacterial growth for all strains tested in contrast to pure HAp spherical granules. The outcomes are consistent with earlier research showing that MgO can inhibit Gram-positive and Gram-negative bacteria [91,92] and that the antibacterial impact relies on MgO concentration. Furthermore, it was clear that *S. aureus* growth was more positively impacted by the HAp/MgO composite’s antibacterial action than *E. coli* growth was. This outcome is consistent with other research that found MgO to have stronger antibacterial activity against *S. aureus* than *E. coli*. Other research, however, demonstrates the reverse result [93]. Their different cell walls, in terms of chemical composition, structure, and thickness, can be associated with differences in antibacterial action toward *S. aureus* and *E. coli.* Gram-positive *S. aureus* bacteria have thick peptidoglycan coatings on their cell walls. Gram-negative bacteria, like *E. coli*, have more complex cell walls with a lipopolysaccharide outer membrane and a weaker peptidoglycan layer, which may give them some resistance to outside dangers [42,94].

In our study, hydroxyapatite samples’ ability to interact with monospecific bacterial biofilm formation was evaluated against *E. faecalis*, *E. coli. S. aureus*, *Ps. aeruginosa* and *C. albicans*. For the samples with cerium, it can be observed that they exhibited an excellent antibacterial effect for *S. aureus* for the concentration of 5 mg/mL, 2.5 mg/mL, 0.62 mg/mL, and 0.15 mg/mL, the hydroxyapatite sample substituted with 5% cerium having the most intense effect, the similar effect is also observed for *E. faecalis* and *C. albicans*. However, in the case of *E. coli,* the hydroxyapatite samples substituted with cerium showed a less antibacterial effect, with little modification that appeared for the sample with 5% cerium. In the case of *P. aeruginosa*, the samples of hydroxyapatite substituted with cerium (1%, 3%, 5%) inhibited the biofilm formation for all concentrations.

In the case of samples of hydroxyapatite substituted with magnesium for *S. aureus*, they inhibited the biofilm formation for the concentration of 5 mg/mL and 2.5 mg/mL for 1%, 3%, 5% magnesium concentration and for the 0.62 mg/mL and 0.15 mg/mL suspension only the sample with 5% magnesium showed an inhibitory effect. In the case of *E. faecalis*, samples substituted with magnesium (1%, 3%, 5%) inhibited the antibacterial activity only for 5 mg/mL and 2.5 mg/mL suspension. For *C. albicans* fungal strain, all three samples of hydroxyapatite substituted with magnesium showed an inhibitory effect for the suspension of 5 mg/mL, 2.5 mg/mL, and 0.62 mg/mL. In the case of *E. coli*, the only inhibition of bacteria can be observed for the sample of hydroxyapatite substituted with magnesium 3% for the suspension of 5 mg/mL and 2.5 mg/mL. As for *Ps. aeruginosa*, the inhibitory effect of bacteria was shown for the suspension of 5 mg/mL for the samples with 1%, 3%, and 5% magnesium concentration, 2.5 mg/mL for the sample with 1% magnesium concentration and for the suspension of 0.62 mg/mL and 0.15 mg/mL all the three samples (1%, 3%, 5% magnesium) shown bacteria inhibitory effect. It can be concluded that the samples of hydroxyapatite substituted with magnesium have a better inhibitory effect for Gram-positive bacteria and *C. albicans* fungal strain.

## 5. Conclusions

The present research pursued the synthesis through the microwave-assisted hydrothermal method and complex characterization of hydroxyapatite substituted with Ce^3+^ or Mg^2+^ powders. Following the analysis results, in all cases, hydroxyapatite was observed as the only crystalline phase, except hydroxyapatite with 5% Mg, for which were also observed the diffraction maxima attributed to the magnesium whitlockite crystalline phase. The size of cerium-substituted hydroxyapatite nanoparticles increased with the Ce^3+^ concentration, while in the case of samples substituted with magnesium, a change in the morphology was observed as the Mg^2+^ concentration increased, acquiring a platelet shape at 5% substitution. Based on the elemental mapping from the EDS analysis, the homogenous presence of the two substituents was confirmed. The FT-IR analysis confirmed the presence of specific groups for the carbonated hydroxyapatite without substantial changes following the addition of the two substituents. At the same time, the values of the hydrodynamic diameter of the hydroxyapatite nanoparticles substituted with cerium and magnesium ions obtained from the DLS analysis showed a high degree of agglomeration that can be observed in the case of all the samples. However, for hydroxyapatite substituted with 5% magnesium, a decrease in hydrodynamic diameter was observed due to the presence of whitlockite. From the biological evaluation, it was confirmed that the obtained hydroxyapatite samples do not produce cytotoxicity, and from the antimicrobial analysis, it was confirmed that the samples inhibit bacteria on *S. aureus*, *E. faecalis*, and *C. albicans* strains. According to microbiological assays, it was highlighted that the obtained samples had an increased antimicrobial effect for Gram-positive bacteria and *C. albicans* and that the cerium-substituted samples may inhibit the biofilm formation better.

## Figures and Tables

**Table 1:** Unit cell parameters, average crystallite size, and crystallinity of hydroxyapatite powders substituted with cerium and magnesium ions at concentrations of 1, 3, and 5% according to Rietveld refinement.

Sample		Unit Cell Parameters						Average Crystallite Size [nm]	Crystallinity [%]
		a [Å]	b [Å]	c [Å]	α [°]	β [°]	γ [°]		
HAp_150		9.431	9.431	6.880	90	90	120	18.65	27.45
HAp_250		9.425	9.425	6.880	90	90	120	46.13	40.49
HAp_Ce_1%		9.444	9.444	6.877	90	90	120	16.15	22.99
HAp_Ce_3%		9.449	9.449	6.873	90	90	120	12.16	21.13
HAp_Ce_5%		9.455	9.455	6.865	90	90	120	10.42	18.31
HAp_Mg_1%		9.446	9.446	6.878	90	90	120	12.81	25.50
HAp_Mg_3%		9.446	9.446	6.876	90	90	120	12.79	26.54
HAp_Mg_5%									
*	HAp 50.1%	9.450	9.450	6.878	90	90	120	12.23	30.99
●	Whitlockit 49.9%	10.419	10.419	37.292	90	90	120	49.54	
